# Where Homœopathy Failed*This article needs to be carefully read to the end before laying the number down, else the reader will fail to understand it.—Editor.

**Published:** 1895-11

**Authors:** A. McNeil

**Affiliations:** San Francisco, Cal.


### WHERE HOMŒOPATHY FAILED.*

A. McNeil, M. D., San Francisco, Cal.

After accepting the very flattering invitation of your energetic and enthusiastic Secretary to write a paper for your society I thought of a subject, and I concluded to choose the above.

It is not a new one, for I venture the assertion that every one who advocates Homœopathy according to Hahnemann has often heard the question thrown at him as being the end of all argument: "What do you do when Homœopathy fails?"

I beg your indulgence in recalling some of my personal experiences where it did fail:

I. I was called to see a plethoric German woman passing through the climacteric, who was suffering excruciatingly from colic. I gave two remedies, first one then the other, without success. It was very difficult to obtain an accurate description of her case. Homœopathy (?) had failed.

II. A case of gall-stone colic, first attack. It is unnecessary to give symptoms showing that it was the passage of a biliary calculus or the guiding symptoms. She was very deaf, which made it even more difficult than in the last case to get the totality. Here was another failure.

---

* This article needs to be carefully read to the end before laying the number down, else the reader will fail to understand it.—Editor.

III. A horse, fine looking, the pick of the stable, had passed through the epizootic, leaving him stiff. It was only with difficulty that he could be backed out of his stall.

After a little exercise he limbered up, so as to travel quite well. I gave him a dozen powders of three grains each made by pouring Rhus-tox., mother tincture, on sugar of milk till it was fully saturated, and then trituating until dry. A week or more passed and no change. Still another failure!

IV. I treated a baby in arms for remittent fever, which changed into an intermittent, perhaps because of my treatment —perhaps not. Two or three weeks passed. Still no better, although I had given different medicines, all in the 30th or 200th.

V. This case, intermittent fever in the fall of the year in Michigan, in the beginning of new practice. A woman of culture who had long been under homœopathic treatment. But yet I failed to even suppress the paroxysms, although I gave Quinine, 15 grains, between paroxysms. Cold weather coming on arrested the disease.

VI. I recently treated a vigorous baby a year old. He had asthma of Millar or larynismus stridulus setting in after convulsions. He had red cheeks with bluish white about the mouth, ground his teeth in his sleep, picked and bored his nose. Cina, from the 40th up to the very top, did no good. But the symptoms changed, so that his mother was afraid of letting him sleep, for as soon as he did a paroxysm of suffocation came on that seemed as if it would end fatally. Of course, I gave Lachesis, and in different potencies from the 200th up, and yet the baby was getting worse and worse, blood becoming carbonized, etc., in short, a miserable failure!

VII. A woman of about thirty years called on me with chronic cough, emaciation, etc. She had been examined by a homœopath whose name many of you would recognize, who said that she had consumption and that she would not live three months.

VIII. I was poisoned with poison oak, which is very frequent in the hills of California. It was the first case of the kind I had had in the State. I took, according to the practice of most of the homœopaths of this coast, Rhus-tox. high, waited twenty-four hours or more. No better. In the East I had always been successful with Graphites$^{cc}$, as prescribed by that fine prescriber, John C. Morgan. It did no good. Next Sepia, as recommended by that prince among homœopaths, the lamented Carroll Dunham. Same result. I then took Anacardium, which is recommended as being specific for cases with certain symptoms by Hering. But still a miserable failure, although I had followed the advice of so many eminent men.

IX. I was called to consult with a homœopathic physician in a case of diphtheria in a two-year-old child.

After entering the room I looked for the attending physician, and not seeing him I inquired and was told that he had gone away, saying, the patient could not live twelve hours. Nevertheless, I was asked to examine the case. The pseudo-membrane was not only in the throat, but the nostrils were full so that it could be seen, and the poor baby was struggling for breath. Had passed urine only once in twenty-four hours. Had eight convulsions, and all this, notwithstanding three homœopathic remedies, one of which I recognized as Mercurius-bin-iod. by its color and two others *smelling* strong. And yet with all this Homœopathy had failed!

X. The first five years of my practice croup was to me a terror; speaking from memory, half my patients died. I read on all the croup remedies, new or old, and gave them in the 30th and 200th, and the destroyer still defied all my efforts. My preceptor, on the other hand, had given tinctures only, and with about the same result. Failure! failure!

Let us see the sequel so that we may find what should be done when Homœopathy fails.

In case I, that of colic, after Homœopathy failed, I sat watching the patient, and when she groaned out: "What shall I do if those cramps come back?" I inquired, what cramp? She replied that she had had frequent attacks like the present, and that there was a liability to cramps in the calves of the legs which were even worse than those in the abdomen which had still continued. I immediately gave a powder of Cuprum$^{30}$.

In a minute she began to talk freely, and she assured me that when that powder was in her throat the pain abated. In five minutes she was free from pain, and, without taking more medicine, continued so for years.

Case II. In this case of gall-stone colic, my patient remarked: "Doctor, how strange it is that every time the pain comes my fingers tingle," and she held out her hands, opening and shutting them. Without further questions I gave her Secale-cor.$^{200}$. In two or three minutes an amelioration was perceptible, which was manifested by the paroxysms becoming shorter and less intense and in an hour she was free from pain. I know the answer will be made, "Oh! yes, the calculus was just ready to pass the duct and to drop into the duodenum." Yes, my doubting Thomas, if that were the case the pain would have ceased instantly and not returned.

But you say there can be no relief as long as the stone is in the narrow tube. You forget, I answer, that it is not the passage through the duct that is painful, but the morbid spasm which occurs in it, and when that is cured the passage may be painless.

III. On careful consideration of the rheumatic condition of the horse I saw, clearly, that Rhus-tox. was the remedy, and because the tincture had failed was no evidence even in a horse that the choice of remedy was wrong, but that more probably the fault was in not giving a potency, so I poured out on his tongue a few pellets of Rhus$^{30}$. In a week or so I inquired about my patient, and learned that he had resumed his former position, viz.: the best horse in the stable, perfectly sound.

IV. On a re-examination of the baby with the intermittent I learned that he had a strong desire to be carried into the open air and that when there he was perfectly contented. Of course, I gave Pulsatilla$^{30}$ and the fever disappeared as if by magic.

V. This is the case of intermittent to which I administered Quinine, 15 grains, between paroxysms without even lessening them. During the winter I prescribed Graphites for an eruption which afflicted her. In the meantime I had sent off and obtained Graphites$^{4000}$ for the herpes. Early in the spring I received an urgent request to call on her. I found her in a chill; she was in despair, and I confess that I soon was in much the same frame of mind, for my only resource, Quinine, had failed. I examined her as well as I could, and taking her eczema into consideration, and as there was no use in returning to Quinine, I gave her Graphites$^{4000}$, and to my astonishment and gratification obtained a fine cure—the first homœopathic cure I made of an intermittent.

Perhaps some of you object to my classifying this case as having homœopathic treatment the first time. I now fully agree with you, but then thought it was, and I fear some men who attach M. D. to their names (authorized to do so by homœopathic colleges) still call such treatment homœopathic.

VI. On studying up my case of Millar's asthma once again, I found the following symptoms, which exactly fitted my case: In Hering's *Guiding Symptoms*, under Hydrocyanic-acid, "Pale bluish face, looks old, pale bluish lips." These symptoms were present in the absence of the paroxysms, and "violent attacks of suffocation, spasmodic cough." At this time the paroxysms were frequent and so violent that death was imminent in each of them. I gave Hydrocyanic-acid$^{30}$. Soon the attacks became less frequent, shorter, and less violent. This progress continued, and was clearly the result of the remedy administered.

VII. In this case of consumption, after a careful questioning, I began with Hepar-sulph.$^{30}$ and went higher. One of the symptoms which guided me was "sensation of a splinter in the throat." Improvement followed immediately, and during the eight years that I had any knowledge of her she remained in fair health.

VIII. Now comes my own case. After I had taken the remedies for my Rhus poisoning I saw that something must be done, as my distress was unendurable. I did then what I ought to have done in the beginning. I carefully studied my symptoms. They were: Intense itching, ameliorated by scratching, so that after rubbing the eruption with a great deal of force with a rough towel I would have a short cessation of the distress; aggravated by cold water so that to wash my hands was something to be dreaded, for by this time the eruption had extended to them. Warmth also aggravated the itching, and I had become extremely irritable. I then worked out my case by Bœnninghausen's *Pocket-Book*. I found that Sulphur led all the other remedies. This I accordingly took in a high potency immediately after the worst attack of itching I had had. There was no itching worth speaking of after, and I made a rapid and satisfactory recovery.

If you wish to cure your patients you cannot do without Bœnninghausen, no other work is comparable with it. And to use it with accuracy and celerity, Yingling's *Checking Lists* are indispensable.

IX. In this case of diphtheria I examined the little patient, and on expressing the opinion that there was a ray of hope, I was asked to take the case. In addition to the symptoms mentioned there were little abraded spots on the face, which the child constantly picked. I gave Arum-tryph.$^{30}$, and my patient fully recovered in a short time.

X. After a period of five years of ill-success in the treatment of croup, I realized that Hahnemann's teaching was true, that as far as therapeutics are concerned that the name of the disease is of no value; or, in other words, the totality of the symptoms is the only guide in the selection of the remedy.

Twenty-two years have now elapsed, and in *all of that time I have not lost a case of croup* of my own patients. I lost one as attending physician after having been given up by another, and one in which I was called in consultation after the case was far gone, and that without changing the potency.

I intended to say before that I have made no distinction between membraneous and spasmodic croup, either in the beginning of my practice or later. I suppose, however, that all the true croup did not get into the first of my practice, or all the false in the latter.

I suppose you have come to the conclusion that it was not Homœopathy that failed, but the homœopa*thist*, at least that is the conclusion I reached.

But I apprehend that some of you would like to ask, "Don't you ever fail?" Oh! yes, often. But as I have shown you in my treatment of croup I did not fail so often after learning that the name of the disease had nothing to do with the treatment, and as I have studied more and learned my materia medica better, and, as more particularly, I learned how to examine patients, and that, as Hahnemann shows, is the principal thing, I do not fail so often, and since I learned to use Bœnninghausen in my difficult cases, I fail still less often.

But if you still persist in asking, *When Homœopathy fails*, what is to be done? What would you think of a hunter who was proudly boasting of the rapidity of fire and the precision of aim of his Winchester, who had carefully concealed about his person a bow and arrow to use when *his repeater failed?* And yet men stand up in homœopathic societies and boast of the superiority of Homœopathy, and have a hypodermic carefully concealed in their vest-pockets, close to their hearts.

This is done in Northern societies. I don't suppose it has ever been done in the South!

Good-bye! till we meet again.

